# Micronutrients in Oncological Intervention

**DOI:** 10.3390/nu8030163

**Published:** 2016-03-12

**Authors:** Uwe Gröber, Peter Holzhauer, Klaus Kisters, Michael F. Holick, Irenäus A. Adamietz

**Affiliations:** 1 Akademie für Mikronährstoffmedizin, Essen, Zweigertstrasse 55, 45130 Essen, Germany; 2 Interdisziplinäres onkologisches Zentrum (IOZ), München, Nußbaumstrasse 12, München 80336, Germany; p.holzhauer@ioz-muenchen.de; 3 Klinik Bad Trissl, Innere Medizin II-Onkologie und Komplementärmedizin, Oberaudorf 83080, Germany; peter.holzhauer@klinik-bad-trissl.de; 4 St. Anna Hospital, Medizinische Klinik I, Herne, Hospitalstrasse 19, Herne 44649, Germany; klaus.kisters@annahospital.de; 5 Boston University Medical Center, 85 East Newton Street M-1033, Boston, MA 02118, USA; mfholick@bu.edu; 6 Klinik für Strahlentherapie und Radio-Onkologie, Ruhr Universität Bochum (RUB), Hölkeskampring 40, Herne 44625, Germany; irenaeus.adamietz@elisabethgruppe.de

**Keywords:** Micronutrients, antioxidants, vitamin D, selenium, l-carnitine, vitamin C, cancer, treatment related side effects, chemotherapy, radiotherapy

## Abstract

Nutritional supplements are widely used among patients with cancer who perceive them to be anticancer and antitoxicity agents. Depending on the type of malignancy and the gender 30%–90% of the cancer patients supplement their diets with antioxidant and immuno-stabilizing micronutrients, such as selenium, vitamin C, and vitamin D, often without the knowledge of the treating physician. From the oncological viewpoint, there are justifiable concerns that dietary supplements decrease the effectiveness of chemotherapy and radiotherapy. Recent studies, however, have provided increasing evidence that treatment is tolerated better—with an increase in patient compliance and a lower rate of treatment discontinuations—when micronutrients, such as selenium, are added as appropriate to the patient’s medication. Nutritional supplementation tailored to an individual’s background diet, genetics, tumor histology, and treatments may yield benefits in subsets of patients. Clinicians should have an open dialogue with patients about nutritional supplements. Supplement advice needs to be individualized and come from a credible source, and it is best communicated by the physician.

## 1. Introduction

According to estimates from the International Agency for Research on Cancer (IARC), there were 14.1 million new cancer cases in 2012 worldwide, of which 8 million occurred in economically developing countries, which contain about 82% of the world’s population. The most common types of malignancy include colorectal cancer, lung cancer, and, depending on gender, breast or prostate cancer. The corresponding estimates for total cancer deaths in 2012 were 8.2 million. By 2030, the global burden is expected to grow to 21.7 million new cancer cases and 13 million cancer deaths simply due to the growth and aging of the population. However, the estimated future cancer burden will probably be considerably larger due to the adoption of lifestyles that are known to increase cancer risk, such as poor diet, physical inactivity, and smoking. Cancers related to these factors, such as breast, lung, and colorectal cancers, are already on the rise in economically-transitioning countries [1,2].

After a long period of stagnation, the conventional medical management of cancer has improved considerably in recent years, with better treatment outcomes and even prolonged survival in some forms of malignancy. New approaches with drug therapy has been mainly responsible for this, but improved diagnostic methods and radiotherapy techniques have also contributed. At the same time, the treatment of cancer has become more intensive and sometimes aggressive, with a corresponding increase in adverse reactions [3,4]. In parallel, the oncology patient’s needs for both gentle therapeutic procedures and complementary measures have increased greatly over the past 15 years. Today, many cancer patients take vitamins and other micronutrients (e.g., selenium, vitamin D) with the aim of improving their standard therapy or reducing the adverse effects of treatment and the underlying disease. Depending on the type of malignancy and the gender of the patient, 30%–90% of people with cancer supplement their diets with antioxidant and immune system stabilizing micronutrients, often without the knowledge of the treating physician [5,6].

Data collected between 2003 and 2010 within the Intergroup Phase III Breast Cancer Chemotherapy trial (S0221) found 48% of patients were taking multivitamins, 20% were taking vitamin C, D, and omega-3 fatty acids, 15% were taking vitamin E, B6, and folic acid, and 34% were taking calcium. Clinicians advised one third to start taking a supplement during treatment, 10% to stop taking one, and 7% to stop all except a multivitamin. 51% of all patients received no advice [7]. From the oncological viewpoint, there are justifiable concerns that dietary supplements decrease the effectiveness of chemotherapy or radiotherapy [5,7,8,9,10]. Recent studies, however, have provided increasing evidence of improved patient compliance, fewer adverse effects and, subsequently, a lower rate of treatment discontinuations, when selected micronutrients (e.g., selenium, l-carnitine, and vitamin D) are added as appropriate to the patient’s medication. There is a better response to the cancer therapy, which in turn improves the prognosis and also the patient’s quality of life [4,11,12,13]. The micronutrients used in complementary oncology have to be selected appropriately and taken at the right time so as not to reduce the effects of the cytoreductive therapy [4,5,11].

There is a need to develop an open and non-judgmental dialogue between oncologists and cancer patients, addressing the needs of the patient while dealing with issues related to the efficacy and safety of micronutrients. Referral of patients to an integrative medicine consultant may help achieve these goals, providing both parties with the option of reaching an informed and respectful decision about treatment. In this review article, we assess the value of specific micronutrients, with an overview of the latest publications and specific recommendations for clinical practice. The review is by no means exhaustive, but presents a selection of the latest findings on important micronutrients used in complementary oncology.

## 2. Malnutrition: The Overlooked Link to Micronutrient Deficiency

The success of treatment and healing processes in people with cancer are greatly influenced by the nutritional status of the patient. This is particularly relevant in clinical practice since, depending on the nature, site, and stage of the malignancy, 30%–90% of patients have an inadequate diet [14,15]. The most severe form of cancer-associated deficiency is seen physically as cachexia. It occurs especially in cases of bronchial, gastric, pancreatic, and prostate cancer (Table 1) [16,17].

An inadequate diet has detrimental effects on the immune status and tolerance of treatment, as well as on various organ and metabolic functions. The effectiveness of measures intended to destroy the cancer (chemo- and radiotherapy) may be reduced, as well as the response to such therapy, while the rate of adverse effects and the risk of treatment-associated complications are increased. This in turn impacts the patient’s quality of life and the prognosis [9,15,17]. Cancer patients with deficient diets have a higher morbidity and mortality: the mortality in malnourished cancer patients is about 30% higher [18,19,20,21,22,23].

**Figure 1:**
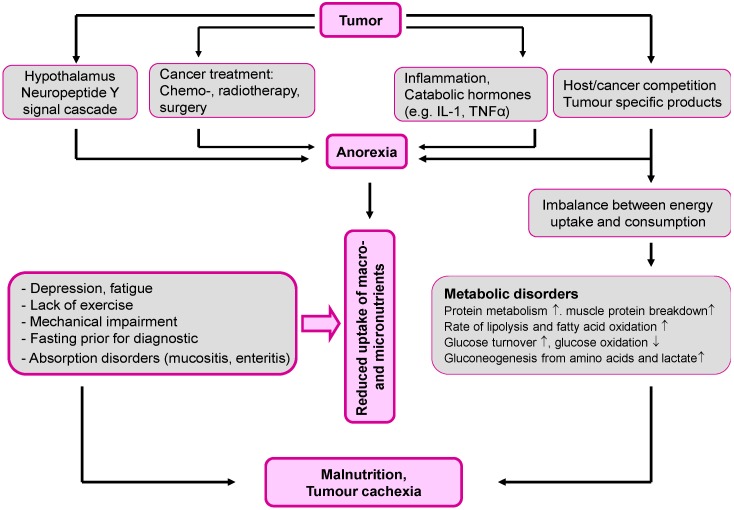
Causes of macro- and micronutrient deficiencies in cancer.

Up to 50% of cancer patients suffer from a progressive atrophy of adipose tissue and skeletal muscle, called cachexia, resulting in weight loss, a reduced quality of life, and a shortened survival time. Anorexia often accompanies cachexia, but appears not to be responsible for the tissue loss, particularly lean body mass [24]. The pathogenesis of cancer-associated malnutrition is multifactorial. In addition to the tumor’s direct consumption, anorexigenic mediators, as well as hormone- and cytokine-induced metabolic disorders also contribute. Pro-inflammatory cytokines (e.g., TNF-α, IL-1β, and IL-6), catabolic hormones (e.g., glucagon and cortisol), and catabolic proteins secreted by the tumors themselves (such as proteolysis-inducing factor, lipid-mobilizing factor, and zinc alpha-glycoprotein), all shift the metabolic equilibrium in the direction of muscle protein and fat breakdown (Figure 1) [4,24,25,26,27].

Malnutrition affects not only the macronutrients that supply energy (carbohydrates, proteins, and fats) but also the biocatalytic and immunomodulating micronutrients. As macronutrients are the natural carriers of micronutrients, malnutrition is one of the main reasons that cancer patients have an inadequate micronutrient status [4,5,26].

According to the European Society for Clinical Nutrition and Metabolism (ESPEN) guidelines on enteral nutrition, it can be assumed that all patients with cancer who consume less than 60% of their daily energy requirements for more than 7–10 days have an inadequate supply of micronutrients [3]. In addition, the consumption and requirements for micronutrients may be increased by the adverse effects of chemo- or radiotherapy (e.g., vomiting, diarrhea, alterations in taste; Table 2) and inflammatory processes. Loss of appetite and aversion to particular foods as a result of anorexia also contribute to a micronutrient deficiency [4,18,27].

## 3. Micronutrient Status in Patients with Cancer

Even at the time of diagnosis and before there are any clinically-relevant changes in the nutritional status, but certainly after the start of treatment, the supply of various vitamins (e.g., vitamin D, vitamin C, and B-group vitamins) and trace elements (e.g., selenium, zinc) is worse in patients with cancer than in healthy people [5,25,28]. Supplies of immunomodulatory and antioxidant micronutrients (e.g., vitamin D, selenium, l-carnitine) and of those with little storage or reserve capacity (e.g., vitamin B1, vitamin C, folic acid, and vitamin K) are particularly critical [27,29,30,31,32,33,34,35,36].

An inadequate supply of antioxidant micronutrients in patients with cancer is reflected, among other things, in raised markers of oxidative stress [29,37,38,39,40,41,42,43,44,45]. Bleeding has also been reported in association with severe zinc deficiency in cancer patients with a poor nutritional status [46].

As cancer- and/or treatment-induced micronutrient deficiency impacts the course of disease and the effectiveness of cytoreductive measures, as well as increasing the risk of complications (e.g., impaired immunocompetence, delayed wound healing, fatigue, and depression), it is necessary to ensure that the patient has an optimal supply of immunostabilizing micronutrients, such as selenium and vitamin D, in addition to an adequate supply of energy substrates (proteins, fats, and carbohydrates). Together with nutrition therapy, the laboratory-validated administration of micronutrients appropriate to the patient’s cancer therapy is, therefore, becoming an important aspect of the concept of adjuvant and complementary oncological treatment [3,4,7,10,11,17,18,47].

## 4. Micronutrient Supplements for Patients with Cancer

Micronutrient deficiencies have, basically, a negative effects on the course of malignant disease and the efficiency of treatment intended to destroy tumors, as it impairs immunocompetence, increases the risk of complications, and impacts on the patient’s physical and mental quality of life [7,18,48,49,50,51,52,53]. The results of several studies have demonstrated the importance of micronutrients as adjuvants to nutrition therapy, and provided evidence that taking a multivitamin/mineral preparation can improve both the quality of life and the prognosis of patients with cancer.

A cohort study of 1129 patients with lung cancer, carried out at the Mayo Clinic (USA), showed that mortality was reduced by 26% in patients taking a micronutrient preparation compared with those not taking any such supplements (95% confidence interval (CI): 0.44–0.65; *p* < 0.01). The mean survival in patients taking micronutrients was 4.3 years compared with two years in patients not taking multivitamin/mineral supplements. Taking a micronutrient preparation was associated with a better quality of life (mean score difference of 3 (95% CI: 0.8–5.1; *p* < 0.01) [52].

In a study on patients with ovarian cancer, who were treated with cyclophosphamide and cisplatin, oral supplements with multiple antioxidants including selenium led to a significant improvement in the immune status and a reduction in the frequency of chemotherapy-induced adverse reactions. In addition to the basic cytostatic therapy, patients (*n* = 31) were given an antioxidant combination consisting of 200 μg selenium, 800 mg vitamin C, 144 mg vitamin E, 60 mg beta-carotene, 18 mg vitamin B2, and 180 mg vitamin B3, in four divided doses, every day for three months. After three months, these patients had significantly higher serum selenium levels (130.23 ± 64.30 compared with 51.41 ± 18.21 μg/L) and greater erythrocyte glutathione peroxidase activity (*p* < 0.0038) than controls who took no antioxidant supplements (*n* = 31). The leucocyte count (e.g., neutrophil granulocytes) was also significantly higher in the group that had taken regular antioxidant supplements (*p* < 0.0001). The incidence of adverse reactions to the chemotherapy, such as loss of appetite, nausea, vomiting, stomatitis, hair loss, flatulence, abdominal pain, weakness, and malaise, was significantly lower in the antioxidant group than in the control group. Neurotoxic symptoms from cisplatin occurred in one woman taking antioxidants and two women in the control group [54].

Antioxidant supplements during cancer therapy are still controversial, as the cytoreductive effects of radiotherapy and some cytostatic agents partially involve the formation of free radicals [7,10,53,55,56]. The vast majority of cytostatics used in treatment today—antimetabolites (e.g., methotrexate), nitrogen mustard derivatives (e.g., cyclophosphamide), platinum complexes (e.g., cisplatin), vinca alkaloids (e.g., vinorelbine), taxanes (e.g., paclitaxel), and anthracyclines (e.g., epirubicin)—do not primarily act through oxidative stress [57,58]. If antioxidants were definitely to reduce the efficiency of standard anticancer therapy, patients would not be allowed to consume any antioxidant- or phytamine-rich fruit and vegetables or green tea (rich in epigallocatechin) during the treatment phase. The often uncritical blanket renunciation of dietary supplements with effective antioxidant and immunomodulating micronutrients during chemotherapy is therefore not justified [59].

Antioxidative micronutrients such as vitamin C, vitamin E, retinoids, and selenium do not act merely as radical scavengers but have many essential metabolic functions in addition to the antioxidant cell protection. They have immunomodulating and apoptosis-inducing properties, as well as regulatory effects on cell proliferation and differentiation. *In vitro*, animal, and human studies have shown that antioxidants reduce cancer cell growth through a variety of mechanisms, including an increase in cell differentiation and apoptosis, as well as the inhibition of protein kinase C and adenylate cyclase activity in neoplastic cells [55,60,61,62].

A systematic review of controlled randomized trials on the effects of antioxidants on chemotherapy concluded that antioxidant supplements do not act detrimentally on chemotherapy, but rather have a beneficial effect on the rate of adverse reactions and the tumor response [63]. None of the studies examined in this review demonstrated a significant impact on chemotherapy. On the other hand, many studies showed that antioxidant supplements increased survival time, the tumor response, or both; they also reduced the rate of adverse reactions in comparison with control groups. These data were confirmed by the results of a further publication from the same group of authors in 2008 [11]. They used a standardized procedure to search the literature published between 1996 and October 2007 in the following databases: Medline, Cochrane, CinAhl, AMED, Alt-HealthWatch, and EMBASE. They included only those randomized controlled clinical trials that had data on the effects of antioxidants on chemotherapy-associated toxicity. Only 33 of the 965 articles examined, with 2446 participants, met the criteria. In particular, evaluation of these trials showed that antioxidant supplements taken during chemotherapy reduced the dose-limiting toxicity of the cytostatic agents, allowing greater tolerability of a higher dose. This working group rightly suggested that further well-designed studies with large numbers of patients and clearly defined antioxidants were necessary to confirm the benefits of adjuvant supplements during chemotherapy [10,11,63,64].

The American Institute for Cancer Research (AICR) recommends that cancer patients undergoing treatment with chemo- and/or radiotherapy should not take multivitamin/mineral preparations that contain antioxidants in a daily dose greater than the corresponding tolerable upper intake level (UL) of the micronutrient concerned (e.g., vitamin C: 2000 mg per day; vitamin E: 250 mg tocopherol equivalent per day). According to the AICR, multivitamin/mineral supplements can generally be regarded as safe when the daily doses of the vitamins and minerals are in the range of the recommended daily allowance (RDA) [18,65,66]. 

Before supplements are prescribed or taken, all attempts are to be made to obtain needed nutrients through dietary sources according to the recommendations of the WCRF/AICR for cancer prevention. We recommend our cancer patients with nutritional problems or weight loss to take a multivitamin/mineral supplement based on the RDA, in order to cover the basic supply of essential micronutrients. This approach primarily compensates for potential deficiencies and is not a high-dose micronutrient therapy. In this article, however, we will review micronutrients, such as vitamin D, selenium, and l-carnitine, which may also benefit patients in supranutritive doses when given appropriately for the specific cancer treatment and based on the results of lab testing.

## 5. Vitamin D

### 5.1. Vitamin D: Sunlight and Cancer

Ecological studies provide the strongest evidence for reduced mortality rates with respect to solar UV-B, with vitamin D production the likely reason. A large body of evidence indicates that solar UV-B (UVB) irradiance and vitamin D reduce the risk of incidence and death for many types of cancer. For men, the UVB index was significantly inversely correlated with 14 types of internal cancer, bladder, breast, colon, gallbladder, kidney, laryngeal, liver, lung, oral, pancreatic, pharyngeal, prostate, rectal, and small intestine cancer. For women, the same UVB index was inversely correlated with bladder, breast, and colon cancer. The results of many studies provide support for the UVB-vitamin D-cancer hypothesis and suggest that the widespread fear of chronic solar UV (UV) irradiance may be misplaced [67,68,69,70,71,72,73,74,75,76,77,78,79,80,81,82,83,84].

### 5.2. Vitamin D: Deficiency and Insufficiency

25(OH)D is the vitamin D metabolite that is measured to assess a patient’s vitamin D status. Vitamin D deficiency is diagnosed when 25(OH)D <20 ng/mL, vitamin D insufficiency is defined as 25(OH)D of 21–29 ng/mL, and 25(OH)D >30 ng/mL is considered sufficient, with 40–60 ng/mL being the preferred range. Vitamin D intoxication usually does not occur until 25(OH)D >150 ng/mL. Vitamin D intoxication is only to be expected at levels of 25(OH)D >150 ng/mL [85,86,87].

It is unrealistic to believe that outdoor activities in the summertime will be able to raise serum 25(OH)D levels to such a significant extent that can be sustained throughout the winter. Typically serum levels of 25(OH)D increase by approximately 10–20 ng/mL by the end of the summer in white Europeans into the range of 35 ng/mL for those exposed to upwards of 300 h of sunshine per month. Since the half-life for 25(OH)D is approximately 2–3 weeks, serum levels decline below the desired 30 ng/mL within 1–2 months after October when sunlight can no longer produce any vitamin D in the skin for those living above 34° north latitude. Vitamin D_3_ supplementation with 2000 IU to 4000 IU vitamin D per day, respectively 40–60 IU vitamin D per kg bodyweight per day will increase serum 25(OH)D levels above 30 ng/mL [85,86,88,89,90]. 

### 5.3. Vitamin D Deficiency in Patients with Cancer

Vitamin D deficiency is common in cancer patients and correlates with disease progression. In observational studies, vitamin D deficiency is associated with increased incidence of breast and colon cancer, as well as with an unfavorable course of non-Hodgkin lymphoma [30,91,92,93,94,95,96]. A recent meta-analysis found a 12% (95% CI = 3%–22%) reduction of lung cancer incidence with respect to 25(OH)D concentrations for an increase from 20 to 50 nmol/L. Another meta-analysis found a relative risk of 0.83 (95% CI = 0.77–0.90; *p* < 0.001) for high *versus* low 25(OH)D concentration [96,97]. 

In both patients with colorectal cancer and those with breast cancer, the vitamin D status has been shown to be an independent predictor of survival [88,98,99,100,101]. There seems to be also an association between vitamin D receptor (VDR) gene polymorphisms and the breast cancer risk. A recent meta-analysis of high-quality studies demonstrated that the Fok1 polymorphism of the VDR gene was closely associated with breast cancer risk [102].

In a placebo-controlled, double-blind study of 1179 postmenopausal women aged over 55 years, the influence of 1400 mg of calcium daily, the combination of 1400 mg of calcium and 1100 IU of vitamin D_3_ or placebo on the cancer risk was studied over a period of four years. In the woman who received the combination of calcium and vitamin D, the 25(OH)D level rose from 28.7 ng/mL to 38.4 ng/mL. Vitamin D status remained unchanged in the two other groups. At the end of the four-year period, the relative risk (RR) of developing cancer was reduced by 60% in the calcium + vitamin D_3_ group as compared with the placebo group relative risk (RR) cancer (RR: 0.402, CI: 0.20, 0.82; *p* = 0.013), while in the group with calcium alone it was reduced by 47% (RR: 0.532, CI: 0.27, 1.03; *p* = 0.063). A reevaluation using logistic regression to cancer-free survival at 12 month showed that the relative risk in the calcium + vitamin D_3_ group had been significantly reduced by 77% (RR 0.232, CI: 0.09, 0.60, *p* < 0.005). The data in the calcium group alone remained virtually unchanged (RR: 0.587, CI: 0.29, 1.21; *p* = 0.147) [86,89,103]. 

In a prospective cohort study, Canadian researchers from the Mount Sinai Hospital in Toronto observed the course of disease in 512 women with breast cancer for about 12 years from 1997 to 2008. The average age of the women was 50.4 years at diagnosis. 37.5% of the breast cancer patients had a vitamin D deficiency [25(OH)D <20 ng/mL or <50 nmol/L] when diagnosed. Only 24% of the affected women had an almost normal vitamin D status [25(OH)D >29 ng/mL or 72 nmol/L]. Vitamin D deficiency was associated with the occurrence of more aggressive forms of breast cancer. After 12 years, the risk of a metastasis in women with a vitamin D deficiency was increased by 94% compared with those with normal vitamin D status (hazard ratio (HR) = 1.94; 95% CI, 1.16 to 3.25). The probability of premature death due to the disease rose in the presence of a vitamin D deficiency by 73% (HR = 1.73; 95% CI, 1.05–2.86) [86,95].

A recent meta-analysis was performed of five studies (*n* = 4443) of the relationship between 25(OH)D and mortality from breast cancer. Higher serum concentrations of 25(OH)D were associated with lower case-fatality rates after diagnosis of breast cancer. Specifically, patients in the highest quintile of 25(OH)D (~30 ng/mL) had approximately half the death rate from breast cancer as those in the lowest (~17 ng/mL) [88]. In another recent meta-analysis that evaluated eight prospective cohort studies from Europe and the USA with over 26,000 men and women (age: 50 to 79 years) and was carried out by the German Cancer Research Centre in Heidelberg an analysis of cancer mortality showed that cancer patients with a 25(OH)D ≤4 ng/mL (≤10 nmol/L) had a 1.7-fold increased risk of dying from the disease compared to cancer patients with a 25(OH)D ≥36 ng/mL (≥90 nmol/L) (risk ratio: 1.70; 95% CI 1.00–2.88) [89,100].

Basal serum 25(OH)D concentrations were retrospectively analyzed in a cohort of melanoma patients (*n* = 324) and healthy controls (*n* = 141) and the hypothesis was tested that serum 25(OH)D concentrations are predictive of melanoma risk, thickness of primary melanomas, and overall survival (OS). Median serum 25(OH)D concentrations were significantly lower (*p* = 0.004) in melanoma patients (median = 13.6 ng/mL) as compared to controls (median = 15.6 ng/mL). Primary tumors of patients with low serum 25(OH)D concentrations (<10 ng/mL) had significantly (*p* = 0.006) greater Breslow thickness (median: 1.9 mm) as compared to patients with higher levels (>20 ng/mL; median: 1.00 mm). Patients with 25(OH)D serum concentrations in the lowest quartile had inferior overall survival (median: 80 months) comparing with the highest quartile (median: 195 months; *p* = 0.049). These data support the concept that serum 25(OH)D concentrations are associated with risk and prognosis of melanoma [104].

### 5.4. Vitamin D and Cancer Therapy

In breast cancer patients under polychemotherapy with anthracycline and taxane, a significant drop in 25(OH)D levels was observed [105]. Some cytostatics (e.g., cyclophosphamide, paclitaxel) are ligands of the pregnane X receptor (PCR) and can, therefore, increase the enzymatic degradation of 25(OH)D and 1α,25(OH)2D via the induction of 24-hydroxylase in the course of chemotherapy [106,107,108]. This mechanism discussed by various authors that explains the abnormalities in bone metabolism under medication is the activation of pregnane X rececptor by some drugs, that may be responsible for the acceleration of vitamin D catabolism through the upregulation of CYP3A4 and CYP24A1, leading to vitamin D deficiency and, eventually, to osteopenia or osteomalacia. Human PXR (hPXR) is also named steroid and xenobiotic receptor (SXR). CYP24 is a multifunctional 24-hydroxylase and the major vitamin D catabolic enzyme that directs the side-chain oxidation and cleavage of 1α,25(OH)2D and 25(OH)D to catabolic carboxylic acid end products. Docetaxel is a known trigger for cutaneous adverse reactions and taste disorders. A vitamin D deficiency can lead to the occurrence of chemotherapy-induced mucositis and dysgeusia. There have been case reports of mucocutaneous side effects (e.g., stomatitis) and taste disorders occurring in cancer patients under polychemotherapy with TCH (T: docetaxel, C: carboplatin, H: trastuzumab), or FOLFOX6, which could be treated successfully with supplementation of vitamin D_3_ [106]. Additionally, arthralgias and fatigue during treatment with aromatase inhibitors, such as letrozole, were significantly reduced by supplementation of vitamin D_3_ (e.g., 50,000 IU vitamin D_3_/week for 12 weeks, PO) in breast cancer patients with vitamin D deficiency [109,110]. 

Similar results are on record for use of bisphosphonates. The osseous effectiveness of bisphosphonates is improved by an adequate vitamin D status (25(OH)D ≥33 ng/mL). This could be related to the fact that a cessation of the parathyroid hormone increase is not achieved until a 25(OH)D level ≥40 ng/mL is reached [111]. Necrotic bone exposure in the oral cavity has recently been reported in patients treated with nitrogen-containing bisphosphonates as part of their therapeutic regimen for multiple myeloma or metastatic cancers to bone. It is suggested that the pathophysiologic mechanism(s) underpinning osteonecrosis of the jaw may involve the interaction between bisphosphonates and compromised vitamin D functions in the realm of skeletal homeostasis and innate immunity [112,113]. In a recently published case-control study with 43 patients, 77% of patients with BRONJ were osteomalacic compared with 5% of patients without BRONJ, according to histomorphometry (*p* < 0.001). Osteomalacia represents a new and previously unreported risk factor for the development of bisphosphonate-related osteonecrosis of the jaw (BRONJ). Due to the high incidence of vitamin D deficiency and the low accuracy of clinical risk factors to predict vitamin D deficiency, screening for vitamin D deficiency before administration of bisphosphonates such as zoledronate may be appropriate. In vitamin D deficiency (until it is corrected) oral bisphosphonates should not be used [114,115,116].

Vitamin D deficiency is common among palliative cancer patients and has been connected to an increased risk for pain, infections and depressions. In a recent prospective, observational study in palliative cancer patients (*n* = 100) low 25(OH)D-levels were associated with a significant increased opioid consumption in palliative cancer patients (*p* = 0.02). A univariate cox regression analysis showed also a weak correlation between survival time and 25(OH)D levels (*p* < 0.05) [117].

Vitamin D deficiency is a risk factor for elderly patients with diffuse large B-cell lymphoma (DLBCL) treated with rituximab, cyclophosphamide, doxorubicin, vincristine, and prednisolone (R-CHOP). In a recent study the impact and mechanisms of vitamin D deficiency on the outcome of elderly patients with diffuse large B-cell lymphoma was investigated. 359 pretreatment 25(OH)D serum levels from the RICOVER-60 study (six *vs.* eight cycles of biweekly CHOP-14 with or without rituximab in elderly patients with aggressive CD20+ B-Cell lymphomas) and 63 from the RICOVER-noRTh study (an amendment to the RICOVER-60 study in which patients received six cycles of cyclophosphamide, doxorubicin, vincristine, and prednisone administered at an interval of two weeks, plus two cycles of rituximab (R-CHOP-14), but without radiotherapy) were determined by chemoluminescent immunoassay. Rituximab-mediated cellular cytotoxicity was assessed by lactate dehydrogenase release assay of CD20+ Daudi cells. RICOVER-60 patients with severe vitamin D deficiency (25(OH)D ≤8 ng/mL) and 25(OH)D levels more than 8 ng/mL treated with rituximab had three-year event-free survival of 59% and 79% and three-year overall survival of 70% and 82%, respectively. These differences were significant in a multivariable analysis adjusting for international prognostic index risk factors with a hazard ratio of 2.1 (*p* = 0.008) for event-free survival and 1.9 (*p* = 0.04) for overall survival. Event-free survival was not significantly different in patients with 25(OH)D levels ≤8 or more than 8 ng/mL (HR, 1.2; *p* = 0.388) treated without rituximab. This was confirmed in an independent validation set of 63 RICOVER-noRTh patients. Rituximab-mediated cellular cytotoxicity increased significantly (*p* < 0.001) in seven of seven individuals with vitamin D deficiency after substitution and normalization of their vitamin D levels. That vitamin D deficiency impairs Rituximab-mediated cellular cytotoxicity and substitution of vitamin D improves Rituximab-mediated cellular cytotoxicity strongly suggests that vitamin D enhances rituximab efficacy [118].

One vitamin D mechanism not widely discussed is the reduction of cancer cachexia, which is characterized by systemic inflammation, weight loss, body-fat atrophy, and muscle wasting. Up to 50% of cancer patients suffer from cancer cachexia and up to 30% may die from it. Several mechanisms associated with cancer cachexia involve cytokines, such as interleukin 1 (IL-1), IL-6, and tumor necrosis factor-α. Vitamin D affects many of these factors, especially those associated with inflammation. IL-6 seemed to be a key mediator of muscle wasting in cancer cachexia. IL-6 is one cytokine that vitamin D suppresses. Vitamin D regulates also the hepcidin-ferroportin axis which may facilitate the bioavailability of iron. Therefore it could also be of importance in the treatment of cancer anemia [18,19,20,21,24,119,120,121,122,123,124].

Radiation-induced injury to normal tissues is a common complication of radiation therapy in cancer patients. Considering the role of vitamin D in mucosal barrier hemostasis and inflammatory responses in a recent prospective observational study it was investigated if vitamin D deficiency is associated with the severity of radiation-induced acute proctitis in cancer patients. 98 patients (57.1% male) with a mean age of 62.8 ± 9.1 years were studied. Vitamin D deficiency was found in 57 patients (58.1%). Symptoms of acute proctitis occurred in 72 patients (73.4%) after radiation therapy (total received radiation dose of 50 Gy). RTOG grade was significantly higher in patients with vitamin D deficiency than in normal cases (median interquartile range of 2 (0.5–3) *vs.* 1 (0–2), *p* = 0.037). Vitamin D deficiency was associated with RTOG grade of ≥2, independent of possible confounding factors (odds ratio (OR) = 3.07, 95% CI: 1.27–7.50, *p* = 0.013) [125].

Some trials show the benefit of vitamin D supplementation in preventing or treating cancer. However, an alternative method can be used to evaluate the evidence obtained to date: Hill’s criteria for causality in a biological system. The Hill criteria relevant for vitamin D and cancer include the following: strength of association; consistent findings in different populations; temporality; biological gradient (dose–response relation); plausibility (e.g., mechanisms); coherence (no serious conflict with known natural history and biology); experiment (e.g., RCT); analogy. Confounding factors should also be accounted for. Not all criteria need be satisfied to claim causality; however, the more they are, the stronger the case. Researchers have evaluated these criteria for cancer in general, and breast cancer in particular. Readers of this article can evaluate how well they think the criteria have been satisfied now [126,127,128,129].

**Recommendation for clinical practice:**

Vitamin D status should be monitored in all cancer patients and treated by adequate vitamin D supplementation (e.g., 40–60 IU vitamin D per kg body weight per day, 25(OH)D target value: 40–60 ng/mL or 100–150 nmol/L). This applies, in particular to cancer patients with poor nutritional status, treatment with aromatase inhibitors, bisphosphonates, and chemotherapy regimens including anthracyclines, taxanes, and monoclonal antibodies, as well as in cases of muscular or mucocutaneous disorders, fatigue, and cancer-related anemia and cachexia.

## 6. Selenium

### 6.1. Selenium and the Risk of Cancer

Selenium is an essential micronutrient for human health whose biological activities and anti-carcinogenic properties likely result from its incorporation as the 21st proteinogenic amino acid selenocysteine in selenoproteins encoded by 25 separate human genes with roles in cell protection from oxidative stress, redox control, and the inflammatory response. Selenium-dependent glutathione peroxidases and thioredoxin reductases are necessary for optimal function of immune cells by controlling oxidative stress and redox regulation. Specific selenoproteins also have ROS-independent roles in modulating inflammatory responses [130]. Pharmacodynamics data suggest that selenite targets several key cancer-associated signaling pathways and induces multimodal regulated cell death pathways [131,132].

The trace element selenium has a long history as a cancer preventive agent. A few human trials addressing total cancer incidence have been published [133,134,135,136]. Some of these studies show a correlation between low serum selenium levels and increased incidence of mainly breast cancer [137], gastrointestinal cancers [138,139], and prostate cancer [140]. The major effects of supplementation have been observed in the incidences of colorectal-, lung, and prostate cancers along with a drastic decrease in the total cancer mortality by 50% [141]. There is also a clear correlation to dose and the base line selenium status of the study population [141]. In the case of prostate cancer beneficial effects are clear only in populations with a low baseline selenium level and a low intake [142,143,144].

A Cochrane review published in 2011 investigated whether there was a relationship between the selenium supply and the risk of cancer, and also the effectiveness of selenium supplements in cancer prevention [145]. The analysis included 49 prospective non-interventional studies and six randomized clinical trials. The epidemiological studies showed that people with a better selenium supply had a reduced incidence of cancer (OR 0.69; 95% CI 0.53–0.91) and a reduced mortality from cancer (OR 0.55; 95% CI 0.36–0.83). The effects on the incidence of cancer was more pronounced in men than in women (OR 0.66; 95% CI 0.42–1.05 and OR 0.90; 95% CI 0.45–1.77, respectively). The randomized clinical trials, however, did not find any effects of selenium supplements, using either selenium yeast to prevent non-melanoma skin cancers or l-selenomethionine to prevent prostate carcinoma. Although an inverse association between selenium exposure and the risk of some types of cancer was found in some observational studies, this cannot be taken as evidence of a causal relation, and these results should be interpreted with caution [145,146].

A critical observation at this point is that the evaluation of the epidemiological data included studies carried out around the world, while merely two studies were considered in the assessment of the preventative effects of selenium, namely the Nutritional Prevention of Cancer Trial (NPCT; selenium yeast for the prevention of non-melanoma skin cancers) [133] and the Selenium and Vitamin E Cancer Prevention Trial (SELECT; l-selenomethionine for the prevention of prostate carcinoma) [147]. It is known, however, that the SELECT participants from the USA, Canada, and Puerto Rico had a median selenium serum level of about 135 µg/L at the start of the study and were therefore already adequately supplied with selenium at baseline. This is one reason why the expected effects of selenium supplements were not seen [147]. In Europe, including Germany, healthy people have mean selenium serum levels about 84 µg/L, while the levels in cancer patients are often even below 70 µg/L [137]. The results of a long-term non-interventional study with 13,887 adult USA citizens demonstrated that an optimal selenium supply gives serum levels between about 110 µg/L and 130 µg/L [148,149]. Above and below this range there was a tendency towards increased mortality from cancer and increased mortality in general. This supports the view that serum selenium levels that are too high (>150 µg/L) do not provide any long-term protection against cancer, as was also the case in the SELECT study. A relevant secondary result of the NPCT study, which primarily investigated the effects of selenium supplements on the risk of skin cancer, is that not only the overall incidence of cancer but also the overall mortality was significantly lower after selenium yeast supplements (200 µg/day) in comparison with placebo [133]. The incidence of prostate cancer was also reduced in the selenium group (relative risk 0.51; 95% CI: 0.29–0.87) with the greatest effect being seen in the men who initially had the lowest selenium supplies (serum selenium levels <123 µg/L) [143,150,151,152].

**Note:** Against this background, both in healthy patients and in those with cancer, the selenium status should always be checked first whenever supplements are being considered. Only when selenium deficiency has been confirmed should supplements be given until a concentration in the optimal range is achieved (serum selenium level: 130 to 150  µg/L). People whose serum selenium concentration is already 122 µg/L or higher should not supplement with selenium [148,149]. The converse is also important: there are various health benefits, and more importantly, no extra risk, for persons with serum concentrations less than 122 µg/L associated with rising their selenium status (e.g., selenium containing supplement) to 130–150 µg/L, a selenium level associated with minimal mortality [3,4,149].

### 6.2. Use of High-Dose Selenium during Chemo- or Radiotherapy

Five randomized trials have looked at the question of whether the concomitant use of high-dose selenium reduces the toxicity of chemo- or radiotherapy without impairing the main effects of oncological treatment. The five available studies on the use of selenium are all randomized but not blinded or placebo-controlled (Table 3). In a Chinese study by Hu *et al.* (*n* = 41), the concomitant use of high-dose selenium significantly reduced the hematotoxicity and nephrotoxicity of cisplatin in the treatment of various solid tumors; survival rates were not given in this publication [153]. Sieja *et al.* (*n* = 31) gave selenium to accompany chemotherapy with cisplatin and cyclophosphamide in patients with ovarian cancer, and found a significant reduction in hematotoxicity, as well as alopecia; survival data were not given here either [54].

In patients with non-Hodgkin’s lymphomas (*n* = 50) and chemotherapy according to the CHOP (cyclophosphamide, doxorubicin, vincristine, prednisone) regimen, the concomitant use of selenium led to a significant reduction in hematotoxicity, a significant improvement in the remission rate, and in the median overall survival [154,155]. A German study (*n* = 39) in which patients took selenium in parallel to radiotherapy for head and neck cancers showed a significant reduction in the rate of dysphagia in the last week of radiotherapy (week 7) [156]. 

In another German study (*n* = 81) on patients with cervical or uterine cancer, taking selenium at the same time as adjuvant irradiation caused a significant reduction in the rate of radiogenic diarrhea (grade ≥ 2) from 44.5% to 20.5% (*p* = 0.04) [49,157,158]. After 10 years, the disease-free survival in the selenium group was 81.5%, *versus* 82.3% without selenium (*p* = 0.87); the overall survival at this point in time was 58.4% in the selenium group, *versus* 44.8% without selenium (*p* = 0.13). We can conclude, therefore, that the administration of sodium selenite is safe and does not reduce the main biological effects of the oncological treatment. In view of its positive effects on RT-induced diarrhea, we consider Se supplementation to be a meaningful and beneficial adjuvant treatment in Se-deficient cervical and uterine cancer patients while undergoing pelvic radiation therapy. All of the selenium studies found a significant increase in the serum selenium concentrations measured after supplements had been taken.

The safety and efficacy of intravenous administered sodium selenite in cancer patients (*n* = 34, 70% of the patients had lung carcinoma) refractory to cytostatic drugs was assessed in a recent phase I trial (SECAR study). Patients received first line of chemotherapy following intravenous (i.v.) sodium selenite treatment to investigate altered sensitivity to these drugs and preliminary assessment of any clinical benefits. Interestingly, many of the patients in this study responded again to their first line of chemotherapy after i.v. administration of sodium selenite. These results were in support of other findings and indicated that even if sodium selenite in itself might be clinically useful in a subset of cancer patients, its subsequent use with chemotherapy might be therapeutically valuable. Furthermore the findings from this study indicate that sodium selenite might work in three ways against cancer: the antitumor effect by itself, by reversing chemoresistance and by ameliorating toxic effects from chemotherapy. In this trial sodium selenite was safe and well-tolerated when administered up to 10.2 mg/m^2^ under the current protocol [159].

**Recommendation for clinical practice:**

In routine clinical practice—outside of studies—the idea is to counterbalance any deficiencies, wherever possible after performing the relevant lab tests. This seems particularly important for the trace element selenium. The studies presented here indicate that the toxicity of chemo- and radiotherapy can be reduced by raising the serum selenium concentration without impacting the main anticancer effects. This is also the practical experience with sodium selenite in our group (e.g., 1 mg sodium selenite in 100 mL 0.9% NaCl as pre-medication before chemotherapy). Efforts should be made to achieve a target selenium level between 130 and 150 µg/L [3,4,149]. In oncology the selenium salt of choice is sodium selenite. Selenomethionine is incorporated non-specifically into proteins in place of methionine and, therefore, accumulate in organs and tissues.

## 7. l-Carnitine 

### 7.1. l-Carnitine: Biological Functions

l-Carnitine is a naturally-occurring quaternary ammonium compound formed from the essential amino acids l-lysine and l-methionine. The human body needs an adequate supply of iron, vitamin C, pyridoxine, and niacin for its endogenous synthesis, which takes place mainly in the cytosol and in the mitochondrial matrix of liver and kidney cells. l-carnitine is critical for energy generation by mitochondrial ß-oxidation. Corresponding to its key position in fatty acid metabolism and energy balance, the largest quantities of l-carnitine are to be found in tissues that meet their energy requirement primarily though the oxidation of fatty acids (skeletal and cardiac muscle). The most important storage compartment is skeletal muscle [3,4,33].

In addition to its widespread use in competitive sports [160], l-carnitine is also being used more often in complementary oncology. The number of international publications on the subject of l-carnitine and cancer has risen enormously in recent years. Numerous preclinical studies and small-scale clinical studies (mostly not conforming to good clinical practice (GCP)) have been published. Apart from basic questions of physiology, bioavailability, and toxicity, the focus of these studies has been supportive questions such as the reduction of chemotherapy-associated neuro- or cardiotoxicity (e.g., paclitaxel and anthracyclines), as well as the reduction of fatigue in the context of anticancer pharmacotherapy (e.g., cisplatin, ifosfamide) [161,162,163,164,165,166,167,168].

In the studies cited, l-carnitine was used in the form of l-carnitine, acetyl- or propionyl-l-carnitine. In the USA, propionyl-l-carnitine is used particularly in cardiovascular disease. Acetyl l-carnitine has the same properties as l-carnitine but, according to the current data, seems to be more effective in the treatment of neurological disorders and neuropathies. The acetylation may increase the enteral absorption ratio for acetyl l-carnitine and make it more effective in penetrating the blood-brain barrier. The cardioprotective effect of propionyl-l-carnitine should be greater than that of l-carnitine or acetyl l-carnitine, because of the intracellular conversion to succinyl coenzyme A (CoA) and subsequent stimulation of the citric acid cycle. In Germany, it is mainly l-carnitine that is used. This is also the only compound available for infusion. We recommend patients with cancer in a poor nutritional condition and on chemotherapy regimens that can induce a carnitine deficiency to take l-carnitine tartrate as oral supplementation because of its better gastrointestinal tolerability [3,4,33].

l-Carnitine shows stabilising effects on the biomembranes of erythrocytes and cell organelles (mitochondria). A pro-apoptotic effect on colorectal cancer cells has also been described; the authors of this publication assume the underlying mechanism to be an increase in the beta-oxidation of fatty acids within cancer cells, which show disrupted energy metabolism [169]. Given that l-carnitine inhibits pro-inflammatory cytokines and skeletal muscle apoptosis, increases available energy from beta-oxidation and, in the context of chemotherapy, may exacerbate pre-existing l-carnitine deficiency [170,171], investigations into the efficacy of l-carnitine in preventing cachexia, anorexia, and fatigue seem to be indicated.

### 7.2. l-Carnitine Deficiency in Patients with Cancer

A deficiency of l-carnitine has been described in many chronic diseases and especially in cancer. Studies on the subject indicate that up to 80% of patients with advanced malignant disease have a general l-carnitine deficiency, for which the body cannot compensate, as is the case with surgical patients [32,33].

Many causes of l-carnitine deficiency are already known in patients with cancer:

- Nutritive l-carnitine deficiency with an inadequate diet (e.g., deficient in iron, vitamin C, and l-methionine).
- Competition with cytostatics (e.g., competition with anthracyclines for the carnitine transporter OCTN2, which is necessary for the transport of l-carnitine into the cells).
- Disruption of l-carnitine biosynthesis by anthracyclines.
- Increase in renal l-carnitine excretion by cisplatin and ifosfamide; formation of non-physiological l-carnitine esters and, hence, increased l-carnitine excretion via the kidneys.

A deficiency of the micronutrient l-carnitine may play a role in prevalent symptoms in patients with cancer such as fatigue, malnutrition, and depression [3,4,32,33,164,166,172,173,174].

### 7.3. l-Carnitine Depletion by Cisplatin and Ifosfamide

**Figure 2:**
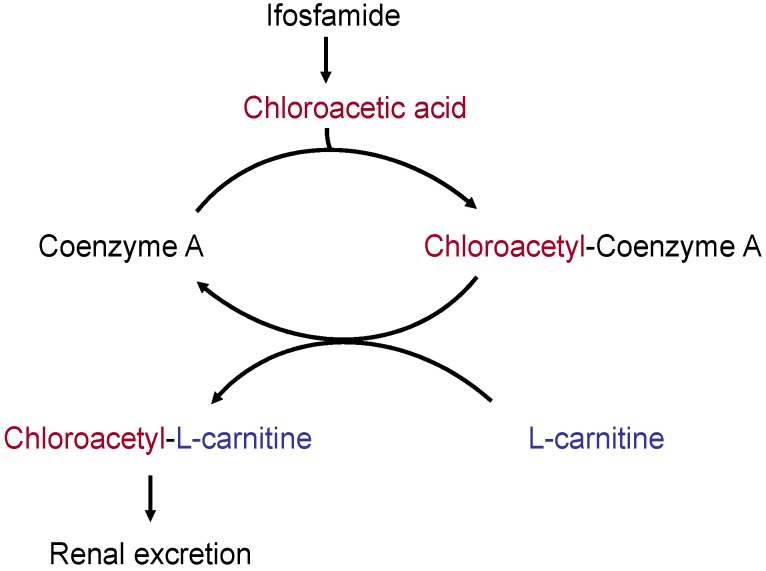
Ifosfamide and carnitine depletion [171].

Cisplatin is nephrotoxic: it inhibits the renal resorption of l-carnitine and can increase l-carnitine excretion in the urine of cancer patients by a factor of 10 [170]. Ifosfamide can also cause considerable renal loss of l-carnitine. Chloracetaldehyde, the metabolite of ifosfamide, may be oxidized to chloroacetic acid, which then binds to free CoA. This in turn disrupts the CoA pool in the mitochondria and inhibits energy-supplying CoA-dependent metabolic pathways. The chloroacetyl group can be transferred from CoA to l-carnitine; in this way it can then be transported out of the mitochondria and out of the cells (Figure 2). As chloroacetyl carnitine is not as well resorbed in the kidneys as free l-carnitine, the excretion of l-carnitine is increased, leading to a secondary l-carnitine deficiency [166,169,174]. 

The consequences of a cisplatin- or ifosfamide-induced l-carnitine deficiency can be:

- l-Carnitine depletion (secondary l-carnitine deficiency in the blood and tissues) with a fall in the plasma l-carnitine levels (<35 µmol/L).
- Impairment of carnitine-palmitoyl transferase 1 activity (an enzyme that catalyzes the carnitine-dependent transport of fatty acids into the mitochondria) and of the cellular carnitine transporter OCTN2.
- Disruption of mitochondrial ATP synthesis, energy deficiency.
- Increased mitochondrial toxicity of cisplatin and ifosfamide.
- Increased risk of fatigue [164] as well as of cisplatin- and ifosfamide-induced adverse neurotoxic and cardiotoxic effects.

l-Carnitine supplements may reduce the cardiotoxic effects of anthracyclines and interleukin-2 and the neurotoxic effects of taxanes or sagolipone, without affecting the cytoreductive effects of these anticancer drugs [32,33,164,175,176,177,178,179,180]. But the results of some trials are still conflicting. An older randomized trial with 30 cancer patients found out that supplementation of l-carnitine (1 g/day) may be used successfully to prevent cardiac complications during interleukin-2 (IL-2) immunotherapy in cancer patients with clinically relevant cardiac disorders. Since cardiac metabolism depends mainly on fatty acid oxidation, the stimulatory role of l-carnitine on fatty acid oxidation could explain at least in part its ability to prevent heart disturbances in response to IL-2 administration [181]. However, a randomized, placebo-controlled trial with 40 patients with non-Hodgkin lymphoma found no evidence that supplementation of l-carnitine (3 g before each chemotherapy cycle, followed by 1 g/day during the following 21 days) protects against anthracyclin-related cardiotoxicity [182]. There is also evidence from two prospective, uncontrolled studies including 52 patients with different grades of CIPN that acetyl-l-carnitine is beneficial in the treatment of paclitaxel- and cisplatinum-induced peripheral neuropathy [161,183]. Evidence that acetyl-l-carnitine or l-carnitine might protect from paclitaxel- and cisplatinum-induced peripheral neuropathy (CIPN) comes from two randomized controlled trials and is contradictory. The findings from the most recent trial, including 409 women receiving adjuvant taxane-containing chemotherapy, introduce a note of caution in that there are indications that acetyl-l-carnitine could even increase CIPN (Table 4) [184]. However the authors of an unpublished double-blind, placebo-controlled trial with 239 cancer patients treated with taxol alone or in combination with other neurotoxic or non-neurotoxic drugs mentions in the abstract a significant action of acetyl-l-carnitine (3 g ALC/day) in improving vibratory sensation in patients with CIPN, compared with placebo was found [185].

### 7.4. High-Dose l-Carnitine Supplements in Patients with Cancer

A deficiency of l-carnitine has been proposed to be an underlying cause of cancer cachexia and tumor associated fatigue [33,165,188]. A few studies have shown that the adjuvant administration of l-carnitine at doses between 2 g and 6 g per day may reduce the weight loss and/or the weakness and fatigue experienced by patients with cancer, but the results of some studies are still conflicting [42,168,186,187,189]. An improvement in the physical and mental health of cancer patients after the administration of high-dose l-carnitine can be attributed not only to an improved cellular energy balance from improved mitochondrial fat burning but also to the favorable effect of l-carnitine on glucose utilization and cytokine metabolism [33,166]. There is evidence from four recent randomized trials, involving more than 800 participants with sufficiently robust measures of cancer-related fatigue that l-carnitine does not reduce moderate to severe cancer-related fatigue or that acetyl-l-carnitine prevents from cancer-related fatigue occurring during taxane-based chemotherapy [33,186,189,190]. These findings contradict those from several uncontrolled studies suggesting that l-carnitine might help against cancer-related fatigue (Table 4) [165,187,191]. There is evidence that l-carnitine has beneficial effects on parameters related to cancer anorexia-cachexia syndrome (CACS) based on the results of a prospective, uncontrolled study including 12 patients with advanced cancers and on the results from one randomized controlled trial [33,191]. In this trial from our group (CARPAN study), which enrolled 72 patients with advanced pancreatic cancer, we found a significant positive effect on both quality of life and nutritional status with l-carnitine therapy (2 g twice daily, p.o.) compared with placebo. We screened 152 and enrolled 72 patients suffering from advanced pancreatic cancer in a prospective, multi-center, placebo-controlled, randomized and double-blind trial to receive oral l-carnitine (4 g) or placebo for 12 weeks. At entry patients reported a mean weight loss of 12 ± 2.5 (SEM) kg. During treatment body-mass-index increased by 3.4% ± 1.4% under l-carnitine and decreased (−1.5 ± 1.4%) in controls (*p* < 0.05). Moreover, nutritional status (body cell mass, body fat) and quality-of-life parameters improved under l-carnitine. There was a trend towards an increased overall survival in the l-carnitine group (median 519 ± 50 days *versus* 399 ± 43 days, not significant) and towards a reduced hospital-stay (36 ± 4 days *versus* 41 ± 9 days, not significant). While these data are preliminary and need confirmation they indicate that patients with pancreatic cancer may have a clinically-relevant benefit from the inexpensive and well tolerated oral supplementation of l-carnitine [33]. 

Cachexic patients can also benefit from a combination of l-carnitine with omega-3 fatty acids. Omega-3 fatty acids exert anti-inflammatory effects and, therefore, recent studies investigated their role in cancer prevention, in cancer cachexia treatment and in enhancement of anti-tumour therapies. Combination of chemotherapy (e.g., gemicitabine) and omega-3 supplementation (e.g., 1–2 g eicosapentaenoic acid/ docosahexaenoic acid (EPA/DHA) daily) appears an effective strategy to enhance the clinical outcome of cancer patients in their curative and palliative clinical trajectory [192,193,194]. 

**Recommendations for clinical practice:**

There is weak evidence from some randomized controlled trials that l-carnitine has a positive influence on cancer anorexia-cachexia syndrome (CACS). Oral supplements and/or parenteral administration of l-carnitine may be of help in cancer patients in a poor nutritional condition and on chemotherapy regimens that can induce a carnitine deficiency, such as cisplatin and ifosfamide. Acetyl-l-carnitine or l-carnitine could also be of help in the prevention and treatment of chemotherapy-induced peripheral neuropathy (e.g., taxanes). 

## 8. Vitamin C

### 8.1. Vitamin C Deficiency

In addition to selenium, vitamin C is the antioxidant micronutrient most frequently used in complementary oncology. Vitamin C deficiency, which may extend to overt scurvy, is found particularly in patients with advanced malignant disease. Pre-existing vitamin C deficiency may be exacerbated by anticancer treatment such as aldesleukin (recombinant interleukin 2) therapy for metastasizing renal cell carcinoma. In patients with cancer, low plasma vitamin C levels (<11 µmol/L) are associated with increased inflammatory activity (raised C-reactive protein (CRP) levels), poor nutritional condition (low albumin levels), and a shorter survival time. There is some evidence that vitamin C potentiates the actions of certain cytostatic agents and, at the same time, attenuates their adverse effects [34,195,196,197,198].

### 8.2. Preclinical Data: Combination with Chemotherapy and Radiotherapy

*In vitro* studies have shown that vitamin C increases the cytotoxic effects of cisplatin, dacarbazine, doxorubicin, paclitaxel, tamoxifen, and fluorouracil [199,200,201]. Animal studies have demonstrated that the intraperitoneal administration of vitamin C together with vitamin K potentiates the cytoreductive effects of various cytotoxic drugs [202]). In addition, combining the administration of doxorubicin with a parenteral dose of vitamin C (2 g/kg body weight, i.v. or intraperitoneal) in animals brings about a significant reduction in the adverse cardiotoxic effects of anthracycline and a significant prolongation of the survival time, while the anthracycline’s cytotoxic effects were not affected [203].

### 8.3. Vitamin C: Effect on Cancer Cells in Vitro 

Further *in vitro* and animal studies have shown that the administration of high-dose vitamin C alone can kill selective cancer cells (ovarian cancer, pancreatic cancer, and glioblastoma) by inducing free radicals, without harming healthy cells [204]. As established by seminal studies, in concentrations higher than 1 mM, ascorbate can cause a build-up of hydrogen peroxide (H_2_O_2_), which is preferentially toxic toward tumor cells. High-dose intravenous vitamin C induces the formation of H_2_O_2_ radicals. Even though these radicals are neutralized in the bloodstream with the help of glutathione peroxidase, this mechanism does not work in the interstitial spaces. Cancer cells are more sensitive than healthy cells to increased concentrations of peroxide radicals [205,206,207].

**Figure 3:**
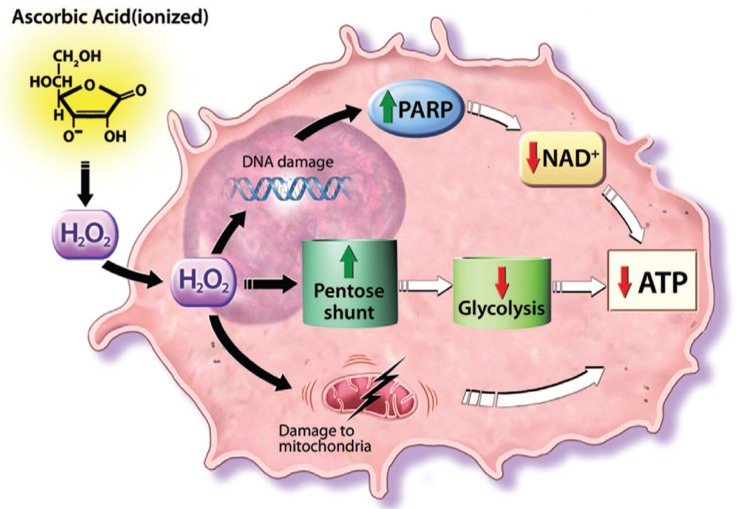
Hydrogen peroxide-dependent cytotoxic effects after ascorbate exposure, according to [204].

It is proposed that external peroxide (H_2_O_2_) radicals formed from pharmacologic ascorbate concentrations diffuse into cells and mediate toxicity in sensitive cells by ATP depletion via one or more pathways (Figure 3). H_2_O_2_ may cause DNA single-strand breaks, repaired by polyADP-ribose polymerase (PARP). Enhanced PARP activity may deplete NAD+, resulting in ATP depletion. On the other hand H_2_O_2_ removal within cells may be mediated in part by glutathione (GSH) peroxidase. GSH peroxidase has an essential requirement for GSH, which, upon enzyme activity, is oxidized to GSH disulfide (GSSG). GSSG is regenerated to GSH with reducing equivalents from NADPH, which in turn is regenerated from glucose via the pentose shunt. Glucose used to reduce NADP+ to NADPH is not available for ATP generation. In cancer cells that depend on anaerobic metabolism for ATP generation (the Warburg effect), loss of glucose to the pentose shunt may result in decreased ATP, leading to cell death. Furthermore mitochondria in some cancer cells may have increased sensitivity to H_2_O_2_. Mitochondria in such cells may be less efficient at baseline in generating ATP compared with normal cells. Enhanced mitochondrial sensitivity to H_2_O_2_, with or without inefficient generation of ATP at baseline, may result in decreased ATP production. These pathways for ATP depletion induced by H_2_O_2_ are independent, and more than one could be responsible for cell death in sensitive cells. Pharmacologic ascorbate concentrations should not impair normal cells because their primary ATP generation is via aerobic metabolism and because their mitochondria may not be as sensitive to H_2_O_2_ as those in some cancer cells [204,207,208].

### 8.4. Vitamin C and Radiotherapy 

According to a recent *in vitro* study, cells from glioblastoma multiform brain tumors seem to be considerably more sensitive to radiotherapy when high doses of vitamin C are given shortly before treatment sessions. The authors of this study showed that the combination of vitamin C (5 mmol/L) with irradiation (6 Gy) killed significantly more tumor cells by inducing double-strand DNA breaks than did either radiotherapy or vitamin C alone [208,209]. A similar effect was seen in leukemia cells irradiated with 2 Gy [210]. Should clinical trials confirm that high-dose vitamin C in combination with radiotherapy increases the chance of cure, it may well be worthwhile combining the two forms of treatment in radiotherapy-resistant cancers.

### 8.5. Vitamin C in Cancer Therapy

Effects on the efficacy of cancer therapy. As part of a prospective Chinese cohort study, 4877 women (aged 20–75 years) with invasive breast cancer were asked about their consumption of vitamin preparations and followed up for a period of about four years on average. Analysis of the data showed that taking vitamins within the first six months of diagnosis was associated with a reduction in mortality and the risk of recurrence; the effects were dependent on the choice of vitamins and the length of time for which they were taken, although the differences did not always reach significance [12]. Mortality was reduced by 44% and the risk of recurrence by 38% (*p* = 0.009 and *p* = 0.01, respectively) in women who took vitamin C for more than three months. In women who had chemotherapy, the risk was similarly reduced, no matter whether they took the vitamins during the chemotherapy or not. In women who had radiotherapy, however, taking vitamins did not reduce either the mortality or the risk of recurrence.

In a recent epidemiological multicenter cohort study, including 15 gynecologists and general practitioners representatively distributed in Germany, data from 125 breast cancer patients in UICC stages IIa to IIIb were selected. A total of 53 of these patients were treated with i.v. vitamin C (7.5 g) additional to standard tumor therapy for at least 4 weeks (study group) and 72 without this additional therapy (control group). Main outcome measures were efficacy in regard to outcome and severity of disease- or therapy-induced complaints during adjuvant chemo- and radiotherapy and aftercare. Comparison of control and study groups revealed that i.v. vitamin C administration resulted in a significant reduction of complaints induced by the disease and chemo-/radiotherapy, in particular of nausea, loss of appetite, fatigue, depression, sleep disorders, dizziness, and hemorrhagic diathesis. After adjustment for age and baseline conditions (intensity score before adjuvant therapy, chemotherapy, radiotherapy), the overall intensity score of symptoms during adjuvant therapy and aftercare was nearly twice as high in the control group compared to the study group. No side-effects of the i.v. vitamin C administration were documented [211].

A case report from gynecologists at the University of Kansas describes a good response to cytotoxic chemotherapy (carboplatin, paclitaxel) in two women with advanced epithelial ovarian cancer who took high-dose oral antioxidants in combination with high-dose vitamin C infusion therapy (15–60 g/infusion, daily to twice a month; the combination was well-tolerated [55,212].

At the present time, there are no further data from controlled interventional studies concerning the effects of vitamin C on the efficacy of chemotherapy. Initial results of high-dose vitamin C infusion therapy (125 g vitamin C per infusion) in combination with gemcitabine (PACMAN study) in a small number of patients with pancreatic cancer have shown good tolerability of the cytostatic drug with the vitamin C infusion, as well as a favorable effect on disease progression [213,214]. 

In a recent case report the regression of multiple pulmonary metastases, which originated from hepatocellular carcinoma after treatment with intravenous administration of high-dose vitamin C are reported. A 74-year-old woman presented to the clinic for her cancer-related symptoms such as general weakness and anorexia. After undergoing initial transarterial chemoembolization (TACE), local recurrence with multiple pulmonary metastases was found. She refused further conventional therapy, including sorafenib tosylate (Nexavar). She did receive high doses of vitamin C (70 g), which were administered into a peripheral vein twice a week for 10 months, and multiple pulmonary metastases were observed to have completely regressed. She then underwent subsequent TACE, resulting in remission of her primary hepatocellular carcinoma [215].

**Recommendation for clinical practice:**

Further studies are needed on the efficacy and safety of high-dose vitamin C. The PDQ cancer information summary for health professionals can help to provide comprehensive, peer-reviewed, evidence-based information about the use of high-dose vitamin C in the treatment of people with cancer. It is intended as a resource to inform and assist clinicians who care for cancer patients. It does not provide formal guidelines or recommendations for making health care decisions. This summary is reviewed regularly and updated as necessary by the PDQ Cancer Complementary and Alternative Medicine Editorial Board, which is editorially independent of the National Cancer Institute (NCI). The summary reflects an independent review of the literature and does not represent a policy statement of NCI or the National Institutes of Health (NIH) [216]. Reported complications of intravenous ascorbate are unusual, but include rare cases of hemolysis in patients with glucose-6-phosphate dehydrogenase (G6PD) deficiency and oxalate nephropathy. Adverse effects may also occur in patients with iron overload and renal failure. Before a vitamin C infusion G6PD should always be assessed therefore by laboratory testing. 

Oral supplements or parenteral administration of vitamin C could be considered in cancer patients with poor nutritional condition, with delayed postoperative wound healing, fatigue or cachexia. Even though the first publications are available showing that vitamin C infusions during chemotherapy have beneficial effects on the incidence of adverse reactions and the patient’s quality of life, their use must be regarded critically until such time as further interventional studies provide clear confirmation of the clinical efficacy and safety of vitamin C infusions in oncology patients. Clinicians should have an open dialogue with patients about vitamin C supplements and vitamin C treatment. The advice for a vitamin C treatment needs to be individualized and come from a credible source, and it is best communicated by the physician.

## Figures and Tables

**Table 1:** Incidence of malnutrition in dependence of the tumor [16,18].

Type of Tumor	Proportions of Patients (%)
Pancreatic carcinoma	83
Gastric carcinoma	83
Esophageal carcinoma	79
Carcinomas of head and neck	72
Colorectal carcinoma	55–60
Pulmonary carcinoma	50–66
Prostate carcinoma	56
Mammary carcinoma	10–35

**Table 2:** Specific chemotherapy-induced micronutrient imbalance (selection) [4].

Cytostatic Agent	Micro-nutrient	Mechanism	Possible Consequences
Cisplatin	l-carnitine	Increased renal excretion of l-carnitine	Cisplatin-induced carnitine insufficiency, increased risk of complications (e.g., fatigue)
Cisplatin	Magnesium, potassium	Increased renal excretion of magnesium and potassium	Hypomagnesaemia, hypokalaemia, disorders of lipid metabolism, glucose intolerance, increased nephrotoxicity
Cyclo-phosphamide	Vitamin D	Increased breakdown of calcidiol and calcitriol to inactive metabolites by 24-hydroxylase	Vitamin D deficiency (calcidiol <20 ng/mL), risk of metabolic bone disorders and impaired immunocompetence
Fluorouracil	Vitamin B1	Inhibition of phosphorylation of thiamine to active coenzyme thiamine diphosphate	Risk of cardiac failure, lactic acidosis, neurotoxicity
Ifosfamide	l-carnitine	Increased renal excretion of l-carnitine	Ifosfamide-induced carnitine insufficiency, increased risk of complications (e.g., fatigue)
Methotrexate	Folic acid	Folic acid antagonism	Folate deficiency, homocysteinaemia, mucositis
Paclitaxel	Vitamin D	Increased breakdown of calcidiol and calcitriol to inactive metabolites by 24-hydroxylase	Vitamin D deficiency (calcidiol <20 ng/mL), risk of metabolic bone disorders and impaired immunocompetence
Pemetrexed	Folic acid	Folic acid antagonism	Mucositis, diarrhea, thrombocytopenia, neutropenia, homocysteinaemia

**Table 3:** Studies on the use of high-dose selenium during chemo- or radiotherapy.

Author	Design	Outcomes
Hu *et al.*, 1997 [153]	Patients with various solid tumors and chemotherapy containing cisplatin (*n* = 41) Randomized crossover study; administration of selenium (as seleno-kappacarrageenan) 4 mg/day for four days prior to and four days after chemotherapy in the first or second cycle	With selenium supplements: clearly higher leucocyte counts 14 days after chemotherapy (3.35 ± 2.01 × 10^9^/L *vs.* 2.31 ± 1.38 × 10^9^/L; *p* < 0.05) Less need for granulocyte colony stimulating factor (110.1 IU *vs.* 723.6 IU, *p* < 0.05) Less need for blood transfusion (0 mL *vs.* 62 ± 38 mL, *p* < 0.05)
Sieja *et al.*, 2004 [54]	Patients with ovarian cancer on chemotherapy (cisplatin, cyclophosphamide; *n* = 31): • Selenium 200 µg/day • Control patients not given any selenium preparations	Significant increases in serum selenium levels, and glutathione peroxidase activity in red blood cells (after 2 and 3 months), and in the leucocyte count (3 months); significant reduction in alopecia, flatulence, abdominal pain, weakness, loss of appetite
Asfour *et al.*, 2006/2007 [154,155]	Patients recently diagnosed with non-Hodgkin’s lymphoma (*n* = 50); Randomized, open-label study: • Chemotherapy plus sodium selenite 200 µg/kg/day; • Chemotherapy according to CHOP regimen	Significant fall in tumor marker Bcl-2 in the group taking supplements after 30 days (end value: 8.6 ± 6.9 ng/mL *vs.* 36.9 ± 7.9 ng/mL; *p* < 0.05 for test substance *vs.* placebo); complete response rate 60% *vs.* 40%; median overall survival in patients with complete remission 21.9 ± 1.4 months *vs.* 19.7 ± 2.0 months; *p* = 0.01
Büntzel *et al.*, 2010 [156]	Patients with advanced head/neck cancer and radiotherapy (*n* = 39) Randomized, open-label study: • Group A: with sodium selenite (500 µg on radiotherapy days, 300 µg on the other days; *n* = 22); • Group B: no selenium replacement (*n* = 17)	Dysphagia (difficulty swallowing): 22.7% *vs.* 35.3%; alteration in taste: 22.7% *vs.* 47.1%; dry mouth: 22.7% *vs.* 23.5%; stomatitis: 36.4% *vs.* 23.5%; only the decrease in difficulties swallowing in the last week of radiotherapy was statistically significant
Mücke *et al.*, 2010 [157]	Patients with cancer of the cervix or uterus (*n* = 81) in the radiotherapy phase following surgical removal of the tumor and with a serum selenium concentration below 84 µg/L; randomized, open-label study: • Group A: with sodium selenite (500 µg on radiotherapy days, 300 µg on the other days; *n* = 39) • Group B: no selenium replacement (*n* = 42)	Significantly increased serum selenium concentration in group A at the end of the study; radiogenic diarrhea (grade ≥ 2) at the end of the study 20.5% *vs.* 44.5% (*p* = 0.04); no difference with respect to blood tests, functional status or quality of life

CHOP: cyclophosphamide, doxorubicin, vincristine, prednisone.

**Table 4:** Studies on the use of l-carnitine and acetyl-l-carnitine in cancer.

Author	Design	Outcomes
Iwase *et al.*, 2016 [168]	Women with breast cancer (*n* = 57) and cancer-related fatigue undergoing chemotherapy; intervention: semi-solid, orally administrable dietary supplement containing coenzyme Q10 and l-carnitine; once daily or regular care for 21 days; multi-institutional, randomized, exploratory trial.	Changes in the global fatigue score, GFS, and current feeling of fatigue were significantly different between the intervention and control groups; HADS, EORTC QLQ-C30, and EORTC QLQ-BR23 scores were not significantly different between the two groups
Hershman *et al.*, 2013 [184]	Women with breast cancer (*n* = 409) undergoing adjuvant taxane-based chemotherapy; intervention: Acetyl-l-carnitine 3 g/day for 24 weeks, control: placebo; randomized, two arms, parallel, blinded, placebo control, 24 weeks follow-up	Chemotherapy induced peripheral neuropathy was significantly increased after 24 weeks Functional status increased CrF unchanged
Campone *et al.*, 2013 [180]	Patients with ovarian cancer or castration-resistant prostate cancer and no evidence of neuropathy (*n* = 150), intervention: Sagopilone, SAG (16 mg/m(2)) intravenously over 3 h every 3 weeks) with Acetyl-l-carnitine (1 g every 3 days) or placebo; Prospective, placebo-controlled, double-blind, randomized trial	No significant difference in overall peripheral neuropathy (PN) incidence was observed between treatment arms, but the incidence of grade ≥3 PN was significantly lower in the acetyl-l-carnitine arm in patients with ovarian cancer compared with a placebo.
Kraft *et al.*, 2012 [33]	Patients with advanced pancreatic cancer (*n* = 72), intervention: 4 g l-carnitine/day for 12 weeks; randomized, two arms, parallel, blinded, placebo control, 12 weeks follow up.	BMI increased, nutritional status increased, quality of life increased, cancer related fatigue unchanged.
Cruciani *et al.*, 2012 [186]	Patients with invasive malignancies and moderate to severe fatigue (*n* = 326); intervention: l-carnitine 1 g, twice daily for 4 weeks or placebo; Randomized, two arms, parallel, blinded, placebo control, four weeks follow-up.	Cancer-related fatigue unchanged, pain unchanged, depression unchanged
Cruciani *et al.*, 2006 [187]	Patients (*n* = 27) with various advanced malignancies (stage unclear) and low plasma carnitine levels, no concurrent chemo-/radiotherapy; intervention: l-carnitine, starting dose: 250 mg/day, increments of 500 mg to a maximum target dose of 3 g/day; quasi-experimental (phase I/II), uncontrolled, pre-post test, one week follow-up.	Cancer-related fatigue decreased, depression decreased, and quality of life increased.
Bianchi *et al.*, 2005 [161]	Patients (*n* = 25) with various cancers (stages unclear) during paclitaxel or cisplatinum chemotherapy and chemotherapy-induced polyneuropathy (CIPN) grade II/III; intervention: Acetyl-l-Carnitine 1 g, twice daily for eight weeks; Quasi-experimental, uncontrolled, pre-post test, eight weeks follow-up.	Sensory and motor neuropathy improved (NCI-CTC scale)
